# Ototoxicity monitoring in South African cancer facilities: A national survey

**DOI:** 10.4102/sajcd.v69i1.846

**Published:** 2022-01-19

**Authors:** Katerina Ehlert, Barbara Heinze, De Wet Swanepoel

**Affiliations:** 1 Department of Speech-Language Pathology and Audiology, Faculty of Humanities, University of Pretoria, Pretoria, South Africa; 2 Department of Speech-Language Pathology and Audiology, Faculty of Healthcare Sciences, Sefako Makgatho Health Sciences University, Pretoria, South Africa

**Keywords:** ototoxicity, ototoxicity monitoring, ototoxicity monitoring protocols, cancer, oncology, hearing loss, chemotherapy, platinum-based compounds

## Abstract

**Background:**

National information regarding ototoxicity monitoring practices are limited for patients undergoing chemotherapy in South Africa.

**Objectives:**

To determine (1) the national status of ototoxicity monitoring implemented in private and public cancer facilities, (2) the knowledge and ototoxicity monitoring approaches implemented, and (3) reported challenges.

**Method:**

A descriptive quantitative survey was conducted in public and private oncology units and audiology referral clinics. Private (60%) and public (43%) oncology units that provide platinum-based chemotherapy in South Africa and audiology referral units (54%) were: (1) surveyed telephonically to determine if ototoxicity monitoring takes place; and (2) a self-administered survey was sent to qualifying oncology units and audiology referral clinics.

**Results:**

All public oncology units reported that ototoxicity monitoring only occurs on referral and is not standard practice. All private oncology units indicated that monitoring is on a patient self-referral basis when symptoms occur. Poor awareness of ototoxicity monitoring best practice guidelines was reported by all oncology units and 14% of audiology referral clinics. Audiology referral clinics reported adequate knowledge of ototoxicity protocols although they are not widely used with only 43% following best practice guidelines. The most prominent challenges reported by participants was referral system (67% oncology units; 57% audiology referral clinics), environmental noise (83% oncology units; 86% audiology referral clinics) and the compromised status of cancer patients (67% oncology units; 57% audiology referral clinics).

**Conclusion:**

Ototoxicity monitoring is not routinely implemented across oncology units in South Africa. Multidisciplinary teamwork and a simplified national ototoxicity monitoring protocol may improve hearing outcomes for patients.

## Background

Cancer is known to be one of the world’s most life-threatening diseases, resulting in approximately 19.3 million new cases and 10 million deaths in 2020 (Sylla & Wild, 2012; World Health Organization, 2020). The projected increase of cancer rates and the progress in cancer therapeutics over the past 40 years, which has remarkably improved survival rates, reveals the need to shift the focus to adverse drug effects and their impact on quality of life (QoL). Ototoxicity is known to have an adverse effect in platinum-based cancer chemotherapeutic agents (Rybak & Ramkumar, 2007; Silver, Baima & Mayer, 2013). Susceptibility to ototoxicity increases with dose and duration of therapy, infusion rate and cumulative lifetime dose, impaired kidney function, which can lead to rapid accumulation of the ototoxic drug, concurrent administration of another ototoxic drug (e.g. aminoglycosides and loop diuretics), anaemia; hypoalbuminaemia; age, pre-existing sensorineural hearing loss, exposure during pregnancy, previous exposure to head and neck radiation, genetic susceptibility and family history of ototoxicity. This in turn has a significant impact on QoL in a cancer survivor’s life (Baguley, Rybak & Ramkumar, 2017; Ferlay et al., 2015; Pearson, Taylor, Patel, & Baguley 2019; Silver et al., 2013).

Platinum-based chemotherapy such as cisplatin is a widely used chemotherapeutic agent for the treatment of numerous malignancies, including testicular, ovarian, bladder, cervical, head and neck and non-small cell lung cancers (Rybak & Ramkumar, 2007). Ototoxicity results in tinnitus and sensorineural hearing loss, which can be severe to profound after high-dose chemotherapy (Rybak & Ramkumar, 2007). For patients with life-threatening illnesses that necessitate treatment with ototoxic drugs, communication ability is a central QoL issue. Hearing loss and tinnitus are both associated with a greater risk of social isolation, depression, anxiety (Nordvik et al., 2018) and development of dementia (Deal et al., 2017). There is also a substantial risk for cochleotoxicity to be followed by vestibulotoxicity in patients receiving platinum-based chemotherapy (Prayuenyong et al., 2018). Vestibular dysfunction may have a major effect on the QoL as balance and mobility impairment are more predominant in cancer survivors, which also increases the risk of falls (Sun, Ward, Semenov, Carey, & Della Santina, 2014; Wildes et al., 2015). Therefore, identifying ototoxic damage early can improve treatment outcomes by minimising hearing loss progression and vestibular dysfunction and providing early aural and vestibular rehabilitation where ototoxicity is inevitable (Konrad-Martin et al., 2018).

Although platinum-based chemotherapy ototoxicity is a common adverse occurrence, there are varying incidence rates reported in both adults and children, which is partly because of the variability of audiological tests employed in the identification and monitoring of the cancer patient’s hearing status (Paken, Govender, Pillay, & Sewram, 2020). Considering these challenges, international bodies such as the American-Speech-Language-Hearing-Association (ASHA) and American Academy of Audiology (AAA) have guidelines that provide flexibility for shortened screening protocols to be used for ototoxicity monitoring (AAA, 2009; ASHA, 1994; Health Professions Council of South Africa [HPCSA], 2018). Although audiologic evaluation is ideally conducted in a sound treated room, the ASHA guidelines recognise that, even with shortened protocols, full booth-based audiometric monitoring is not always feasible in all clinical environments (Brungart et al., 2018), which contributes to the ineffectiveness of existing screening programmes.

It is currently unknown what proportion of patients undergoing chemotherapy with platinum-based agents are systematically identified and monitored for signs of ototoxicity in South Africa. Too often, audiological testing is arranged only once debilitating hearing loss is already apparent to the patient or multidisciplinary team (Paken et al., 2020). Serial audiological monitoring is critical in ototoxicity monitoring protocols to achieve the desired outcomes (Brungart et al., 2018; HPCSA, 2018). Another challenge is that, whilst much chemotherapy practice is protocol based, divergence from protocols is common as treatments may be delayed, modified or added to in particular circumstances (Baguley et al., 2017). This often affects the audiological monitoring schedules, highlighting that set protocols cannot be followed for all patients receiving chemotherapy. Thus, the identification of a truly homogeneous treatment group may be difficult. Whilst empirical evidence of compliance with such guidelines has not been identified, indications are that the implementation of audiometric monitoring is sporadic (Paken et al., 2020).

In low- and middle-income countries (LMICs) like those in sub-Saharan Africa, there is a lack of hearing care and appropriate equipment in order to successfully implement hearing screening and monitoring programmes (Chadha, Cieza, & Krug, 2018; Mulwafu, Kuper, & Ensink, 2016). The number of audiologists within the African continent has been reported to be one of the lowest, with an estimate of one audiologist for every million people in sub-Saharan Africa (Mulwafu et al., 2016). Moreover, the high costs associated with screening equipment and the necessity for the equipment to be operated by trained personnel such as audiologists further burden the implementation of effective screening programmes for early detection and intervention (Louw, Swanepoel, Eikelboom, & Myburgh, 2017). Furthermore, the mechanisms for tracking patients throughout the system need to be explored in order to ensure patients receive the audiological services they may need at various stages of cancer treatment and survivorship (Konrad-Martin et al., 2018).

Studies in South Africa (Andrade, Khoza-Shangase, & Hajat, 2009; Khoza-Shangase & Jina, 2013) revealed that the effects of ototoxicity, the role of audiologists and need for their expertise were not fully recognised by the oncologists. Furthermore, most general practitioners (GP) also do not appear to carry out ototoxicity monitoring strategies despite being aware of their own role within an ototoxicity monitoring programme (Andrade et al., 2009; Garinis et al., 2018; Khoza-Shangase & Jina, 2013).

Early identification of ototoxic effects on hearing ability because of platinum-based therapy provides physicians with an opportunity to adjust the drug therapy in order to minimise or prevent hearing loss and provide early hearing intervention services (Garinis et al., 2018; HPCSA, 2018). An ototoxicity monitoring programme should be context sensitive without increasing the already over-burdened treatment schedule of cancer patients, identify ototoxic effects early and include a team of healthcare professionals (Ganesan et al., 2018). Studies conducted in South Africa indicated that there was neither provision for ototoxicity monitoring in the chemotherapy protocols nor any ototoxicity monitoring programmes in place and only half of the participants reported referring patients for audiological management during the chemotherapeutic process (Khoza-Shangase & Jina, 2013; Paken et al., 2020). The studies that have been performed in South Africa are limited to certain geographical areas and national data on ototoxicity monitoring practices in South Africa is lacking. This study therefore aimed to describe ototoxicity monitoring practices in South Africa in both the private and public healthcare sectors.

## Method

The survey aimed to (1) describe the national status of ototoxicity monitoring implemented in private and public cancer facilities in South Africa, (2) describe knowledge and ototoxicity monitoring approaches implemented and (3) identify challenges to ototoxicity monitoring.

### Data collection sites, population and sampling

Probability sampling was applied. The participants included healthcare professionals (GPs, oncologists, nurses, pharmacists and audiologists) working in private and public healthcare oncology units and audiology referral clinics in South Africa. All public hospitals were accessed via the national department of health website http://www.health.gov.za/ (Department of Health, 2019). Private oncology units were accessed via www.medpages.co.za (Medpages, 2018) and the Independent Clinical Oncology Network (ICON) (ICON, 2017). Public provincial tertiary and central or academic hospitals were included as these hospitals consist of specialised referral units, which together provide an environment for multi-specialty clinical services, innovation and research, such as oncology. There are 29 tertiary hospitals and 10 major teaching hospitals in South Africa; however, not all hospitals provide platinum-based oncology treatment or were able to participate in the research. There were 55 private oncology units identified in South Africa. Oncology units were contacted telephonically to confirm eligibility and willingness to participate. Information was obtained from the practice manager or nurse in charge. Once consent for participation was obtained, questionnaires were sent to the oncology units where a healthcare professional (GPs, oncologists, nurses or pharmacists) representing the oncology units completed the questionnaire.

**FIGURE 1:**
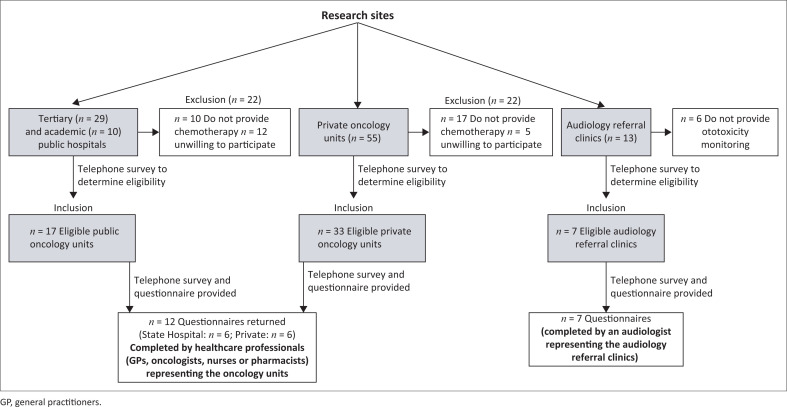
Research sites, participant description and sampling procedure.

Audiology referral clinics (*n* = 13) in the same hospital as oncology units were contacted for participation in the study. Participants therefore included an audiologist representing the audiology referral clinics in the private and public sector across South Africa and mentioned by oncology units as referral centres. Questionnaires were sent to the audiology referral clinics for completion. Figure 1 illustrates the research sites, participant description and sampling procedure.

### Data collection procedure

Before commencing with the study, ethical clearance was obtained from a university in South Africa.

Firstly, private oncology units (*n* = 55) and public hospitals (*n* = 29) with cancer units were contacted telephonically to determine whether platinum-based chemotherapy is being offered as a treatment option. Oncology units who offered platinum-based chemotherapy and were willing to participate in the study were surveyed telephonically. During the telephonic survey, nursing managers and oncologists in the units provided information regarding ototoxicity monitoring practices within the cancer units. The telephonic survey confirmed (1) if platinum-based chemotherapy agents are offered in the unit, (2) if ototoxicity monitoring is performed as standard practice for all patients receiving ototoxic chemotherapy, if ototoxicity monitoring is only performed when referred by a healthcare professional or if patients arrange their own hearing evaluation when ototoxicity symptoms or hearing loss is apparent and (3) where patients are referred for ototoxicity monitoring. The second part of the research study included a self-administered questionnaire. An electronic questionnaire was sent to the oncology units to determine the knowledge, monitoring approaches, protocols and challenges of implementing ototoxicity monitoring. Nurses, oncologists, GP and pharmacists were some of the healthcare professionals who completed the electronic questionnaires on behalf of the oncology units.

Audiology departments in public hospitals with cancer units and private practice audiologists in close proximity to private oncology units and those mentioned as referral centres were contacted for information on ototoxicity monitoring practices. The telephonic survey confirmed if ototoxicity monitoring is performed for patients receiving ototoxic chemotherapy. The electronic questionnaire was sent to the identified audiology referral clinics for completion. Audiologists completed the questionnaire on behalf of the audiology referral centres.

### Description of electronic questionnaire

A structured, self-administered questionnaire was used for both oncology units and audiology referral clinics. The questionnaire was administered as a single attempt to determine the characteristics of the ototoxicity monitoring protocols currently implemented in oncology units and audiology referral clinics in South Africa and the challenges experienced. The same questionnaire was used for all healthcare professionals as the questionnaire included general aspects regarding ototoxicity, ototoxicity monitoring and challenges. There was a section only to be completed by audiologists that included aspects such as testing protocols and procedures followed during ototoxicity monitoring. The questionnaire was adapted from Steffens et al. (2014) by adding answer options to choose from, resulting in more closed-ended questions with an option of providing additional information. The original study (Steffens et al., 2014) had only open-ended questions, which were interview based.

The questionnaire included a range of open- and closed-ended questions (multiple choice format) in three broad categories: (1) demographic information, (2) knowledge and general perceptions towards ototoxicity monitoring, (3) challenges, (4) ototoxicity monitoring protocols (only to be completed by audiology referral clinics) and (5) views on potential improvements to ototoxicity monitoring. Qualtrics survey platform was used for ease of completion and automatic data storage (refer to questionnaire in Online Appendix 1).

### Data analysis

Data collected from the (1) telephonic surveys and (2) electronic questionnaires with private and public oncology units and audiology referral clinics were integrated. The data were analysed to yield percentages and frequency distributions nationally and across provinces. Thematic content analysis was used for open-ended questions.

## Results

From the 39 hospitals in the public sector that provide chemotherapy oncology services, 44% (*n* = 17) were willing to participate in the research following the telephonic survey. From the 55 private oncology units, 60% (*n* = 33) were surveyed telephonically and provided platinum-based chemotherapy; some units only provide radiation or were unwilling to participate. A lower response rate was received for the questionnaire compared with the telephonic survey as some of the units did not perform ototoxicity monitoring and did not consent to complete the questionnaire. Furthermore, some oncology units also have several branches, and responses were only obtained from one branch as similar ototoxicity monitoring practices are followed at the branches.

The electronic questionnaire was completed by 26 (46%, *n* = 57) participants; however, only *n* = 19 (33%, *n* = 57) could be included in the study as they complied with the inclusion criteria. Questionnaires completed by healthcare professionals that are not involved in ototoxicity monitoring and working with oncology units were excluded from the study. Therefore, only 36% (*n* = 12) questionnaires were completed and returned by healthcare professionals representing the oncology units. The questionnaires were completed by 54% (*n* = 7) audiologists representing the audiology units. Overall a response rate of > 25% for completion of questionnaires was achieved, which is considered acceptable for mailed surveys (Baruch & Holtom, 2008).

### Telephonic survey: Ototoxicity monitoring coverage

Telephonic surveys of ototoxicity monitoring at private and public oncology units demonstrated that it was not a standard practice. Cancer patients with ototoxicity complaints, such as hearing loss and tinnitus, were either referred for an audiological evaluation by a healthcare professional or patients had to arrange for audiological evaluations on their own initiative. Table 1 provides a breakdown of the ototoxicity monitoring approaches followed.

**TABLE 1:** Distribution of oncology units and ototoxicity monitoring approaches (*n* = 50) across public and private facilities.

Province	No. of public oncology units	No. of private oncology units	Ototoxicity monitoring approaches	
			Public healthcare: By professional referral†	Private healthcare: Patient self-referrals‡
Gauteng	4	12	4	12
Free State	1	1	1	1
Mpumalanga	1	1	1	1
Limpopo	1	2	1	2
North West	1	2	1	2
Western Cape	4	7	4	7
Northern Cape	1	2	1	2
Eastern Cape	2	2	2	2
KwaZulu-Natal	2	4	2	4

**Total**	**17**	**33**	**17**	**33**

† , Professional referral refers to referral from a healthcare professional within the oncology unit.

‡ , Patient self-referral refers to patient makes own appointment with an audiologist when ototoxicity symptoms or hearing loss is apparent.

### Self-administered questionnaire: Ototoxicity perceptions, challenges and testing approaches

Table 2 summarises the demographic information of the participants (healthcare professionals representing the oncology units and referral audiology centres).

**TABLE 2:** Demographic information of the participants.

Participant demographics	Oncology units percentage (*n* = 12)†		Audiology referral clinics percentage (*n* = 7)‡	
	*n*	%	*n*	%
**Average age (in years)**				
20–25	0	8	5	71
26–30	0	92	0	29
31–35	0	-	2	-
36–40	1	-	0	-
41+	11	-	0	-
**Gender**				
Males	2	17	1	14
Females	10	83	6	85.7
**Years of experience in oncology (years)**				
0–5	2	17	6	86
6–10	1	8	0	0
11–16	3	25	1	14
> 21	6	50	0	0
**Current working place**				
Public	4	33	3	43
Private	8	67	4	57
**Profession**				
General practitioner	2	17	0	0
Nurse	5	42	0	0
Audiologist	0	0	7	100
Oncologist	3	25	0	0
Pharmacist	2	17	0	0
**Ototoxicity knowledge acquired (select all that apply)**				
University programme:	3	25	7	100
On the job:	7	58	0	0
Own reading:	6	50	5	71
Conferences and workshops	4	33	4	57

† , *n* = 12 healthcare professionals representing the oncology units.

‡ , *n* = 7 audiologists representing the audiology referral clinics.

Multiple-choice questions were used to determine general knowledge and perceptions of ototoxicity monitoring. Overall, poor awareness of ototoxicity monitoring protocols or best practice guidelines were reported, as no oncology units were reported to have knowledge about protocols. Amongst the audiology referral clinics, 14% (*n* = 1) had knowledge of best practice guidelines and 86% (*n* = 6) had no knowledge. All participants (100%) from the oncology units and audiology referral clinics described ototoxicity as ‘A side effect of medicine resulting in auditory and vestibular dysfunction resulting in hearing loss and disequilibrium’. The purpose of ototoxicity monitoring was reported as early identification of hearing loss (83%, *n* = 10 oncology units; 86%, *n* = 6 audiology referral clinics), to terminate ototoxic treatment (0%, *n* = 0 oncology units; 14.3%, *n* = 1 audiology referral clinics), to adjust treatment dosages (67%, *n* = 8 oncology units; 86%, *n* = 6 audiology referral clinics), to improve QoL post-treatment (25%, *n* = 3 oncology units; 57%, *n* = 4 oncology referral clinics) and to provide appropriate and timely intervention (83%, *n* = 10 oncology units; 86%, *n* = 6 audiology referral clinics). In contrast, the benefits of providing ototoxic monitoring to the patient was reported as patient knowledge of ototoxic hearing loss (58%, *n* = 7 oncology units; 43%, *n* = 3 audiology referral clinics) and early identification (100%, *n* = 12 oncology units; 100%, *n* = 7 audiology referral clinics) and intervention (83%, *n* = 10 oncology units, 100%, *n* = 7 audiology referral clinics) of hearing loss. Table 3 describes participants’ knowledge of ototoxicity.

**TABLE 3:** Participant’s general knowledge and perceptions of ototoxicity monitoring.

Areas of knowledge in ototoxicity	Oncology units percentage (*n* = 12)†		Audiology referral clinics percentage (*n* = 7)‡	
	*n*	%	*n*	%
**Signs of ototoxicity**				
Hearing loss	12	100	7	100
Disequilibrium	9	75	6	86
Renal impairment	0	0	0	0
**Cancer drugs causing HL**				
Fosfamide	1	8.3	1	14
Cisplatin	12	100	7	100
Methotrexate	0	0	1	14
**Configuration of HL from ototoxicity**				
High frequency hearing loss	6	50	7	100
Unsure	6	50	0	0
**Severity of HL**				
Moderate	3	25	1	14
Severe	1	8	2	29
Profound	1	8	4	57
Unsure	7	58	0	0
**% Patients receiving cisplatin will develop HL**				
1–24	3	25	0	0
25–49	4	33	3	43
50–74	2	17	1	14
75–99	1	8	2	29
100	2	17	1	14
**Likelihood of tinnitus developing**				
Slight likelihood	0	0	0	0
Moderate likelihood	3	25	0	0
Very likely	9	75	7	100
**Likelihood of developing vestibular problems**				
Slight likelihood	3	25	0	0
Moderate likelihood	5	42	4	57
Very likely	4	33	3	43

HL, hearing loss.

† , *n* = 12 healthcare professionals representing the oncology units.

‡ , *n* = 7 audiologists representing the audiology referral clinics.

**FIGURE 2:**
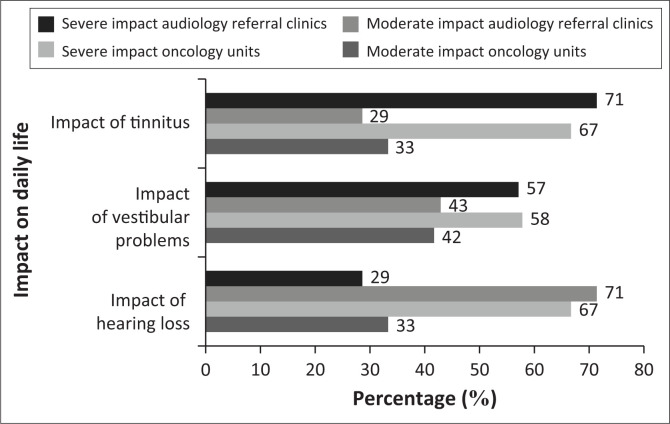
Participant’s perception of the impact of ototoxicity symptoms on daily life (*n* = 12 healthcare professionals representing the oncology units, *n* = 7 audiologists representing the audiology referral clinics).

Participants reported on the severity of the possible impact (tinnitus, hearing loss and vestibular problems) of ototoxicity on cancer patients’ daily life as presented in Figure 2.

The questionnaire probed the ototoxicity monitoring protocols followed when cancer patients attend hearing evaluations and the importance of baseline testing. Baseline testing in ototoxicity monitoring was deemed important as 60% (*n* = 7 oncology referral units), 100% (*n* = 7 audiology referral clinics) reported extremely important and 42% (*n* = 5 oncology units) said very important. However, this does not seem to reflect in practice as the audiology referral clinics reported that only 29% (*n* = 2) of oncology patients receive baseline assessments. All participants (100%, *n* = 12 oncology units; *n* = 7 audiology referral clinics) reported that only patients referred receive baseline assessments and 17% (*n* = 2 oncology units) reported that baseline assessments were not performed. Section 4 (refer to the appendix) of the electronic questionnaire (refer to Online Appendix 1) was completed by audiologists only (*n* = 7 audiology referral clinics). Table 4 describes the battery of audiological tests included in ototoxic monitoring when patients are referred or self-refer for audiological testing.

**TABLE 4:** Battery of audiological tests included in ototoxic monitoring by audiology referral clinics (*n* = 7 audiologists).

Audiological tests	Baseline testing % (*n* = 7)		Serial monitoring % (*n* = 7)	
	*n*	%	*n*	%
Pure tone audiometry (PT)	7	100.0	7	100.0
Extended high frequency audiometry (EHF)	5	71.4	6	57.7
Distortion product otoacoustic emissions (DPOAE’s)	4	57.1	5	71.4
Vestibular assessments	1	14.1	0	0.0
Other (not specified)	2	28.6	1	14.1

The participants were asked how informed patients were regarding the ototoxic effects of chemotherapy: 0% (*n* = 0 oncology units) and 71% (*n* = 5 audiology referral clinics) reported patients were uninformed, 8% (*n* = 1 oncology units) and 29% (*n* = 2 audiology referral clinics) reported slightly informed, 83% (*n* = 10 oncology units) reported moderately informed and 8% (*n* = 1 oncology units) reported well informed. The participants reported that patients receive this information from oncologists (50%, *n* = 6 oncology units; 57%, *n* = 4 audiology referral clinics), nurses (42%, *n* = 5 oncology units; 71%, *n* = 5 audiology referral clinics), audiologists (25%, *n* = 3 oncology units; 100%, *n* = 7 audiology referral clinics), pharmacists (25%, *n* = 3 oncology units; 14.3%, *n* = 1 audiology referral clinics) and GPs (8%, *n* = 1 oncology units; 14.3% *n* = 1 audiology referral clinics). The majority of participants agreed that oncologists (92%, *n* = 11 oncology units; 86%, *n* = 6 audiology referral clinics) and nurses (83%, *n* = 10 oncology units; 86%, *n* = 6 audiology referral clinics) are responsible for informing patients. Audiologists (58%, *n* = 7 oncology units; 57%, *n* = 4 audiology referral clinics), GPs (58%, *n* = 7 oncology units; 57% audiology referral clinics) and pharmacists (50%, *n* = 6 oncology units; 43%, *n* = 3 audiology referral clinics) also have a responsibility to inform patients. Although ototoxicity monitoring is not a standard practice, but rather based on a referral basis, provision of ototoxicity monitoring services was reported by 25% (*n* = 3 oncology units) and 29% (*n* = 2 audiology referral clinics; 42% [*n* = 5 oncology units] and 43% (*n* = 3 audiology referral clinics) stated no ototoxicity monitoring was provided and 33% (*n* = 4 oncology units) and 29% (*n* = 2 audiology referral clinics) reported to be unsure.

From the responses completed only by audiology referral clinics (*n* = 7), only 43% (*n* = 3) reported that the ototoxicity monitoring protocols are documented, and it is the hospital’s protocol of unknown origin, 43% (*n* = 3) were unsure and 14% (*n* = 1) reported protocols are not documented. Only 14% (*n* = 1) reported that the protocols are compulsory and always followed, 29% (*n* = 2) said it was only a guideline and sometimes followed and 57% (*n* = 4) were unsure if protocols are followed. The factors that influence the protocols followed were reported as follows: 29% (*n* = 2) stated testing was performed according to clinical necessity and doctor referrals, 43% (*n* = 3) reported best practice guidelines are followed, 57% (*n* = 4) mentioned availability of equipment, 29% (*n* = 2) stated appointment availability and 57% (*n* = 4) mentioned audiologist training and knowledge as a contributing factor. Audiologist referral units (*n* = 7) reported sending ototoxicity testing and monitoring results to oncologists (71%, *n* = 5) and nurses (14%, *n* = 1) and to the patient (29%, *n* = 2). The results provided were believed to influence dosage choices (86%, *n* = 6), influence treatment choices (57%, *n* = 5), results in otoprotective agents being prescribed (29%, *n* = 2) and all audiology referral clinics (100%, *n* = 7) agreed that it ensures follow-up appointments and frequent visits to the audiologist.

The length of monitoring varied as 50% (*n* = 6 oncology units) and 43% (*n* = 3 audiology referral clinics) reported that monitoring should continue for 12 months whilst 42% (*n* = 5 oncology units) and 43% (*n* = 3 audiology referral clinics) were of the opinion that it should continue for the patient’s lifespan and only 8.3% (*n* = 1 oncology units) and 14.3% (*n* = 1 audiology referral clinics) indicated that 6 months of monitoring is sufficient. Most participants (83%, *n* = 10 oncology units; 100%; *n* = 7 audiology referral clinics) agreed that the audiologist should decide how long monitoring is needed, whilst 17% (*n* = 2 oncology units) indicated the oncologist should decide.

### Challenges to implementation of ototoxicity monitoring

The final section of the questionnaire surveyed the challenges of implementing ototoxicity monitoring in cancer patients. All (100%, *n* = 12 oncology units and *n* = 7 audiology referral clinics) of the participants reported a greater awareness needed amongst health professionals; however, 25% (*n* = 3 oncology units) reported that awareness amongst oncologists is not needed. Participants were asked if improvements are needed in ototoxicity monitoring in their workplace, 50% (*n* = 6 oncology units) and 57% (*n* = 4 audiology referral clinics) reported ‘yes’, 17% (*n* = 2 oncology units) and 14% (*n* = 1 audiology referral clinics) reported ‘no’ and 33% (*n* = 4 oncology units) and 29% (*n* = 2 audiology referral clinics) reported ‘unsure’. Participants were asked if the referral process for ototoxic monitoring posed a challenge, 8% (*n* = 1 oncology units) and 14% (*n* = 1 audiology referral clinics) reported ‘yes’, 33% (*n* = 4 oncology units) and 29% (*n* = 2 audiology referral clinics) reported ‘no’ and 58% (*n* = 7 oncology units) and 57% (*n* = 4) reported ‘unsure’. Open-ended responses in the questionnaire from a referral audiology clinic in the public sector (14%, *n* = 1) reported that ‘an attempt was made to implement a strict ototoxicity monitoring system for all qualifying chemotherapy patients, however this was unsuccessful’. Oncology referral units indicated that ‘at-risk patients or patients with hearing loss complaints, rather than all patients are identified for possible ototoxicity monitoring’. It was also reported that ‘hearing loss does not seem to be a main complaint in patients seen’. The patient challenges experienced were as follows: too ill to attend the audiology clinic (67%, *n* = 8 oncology units; 57%, *n* = 4 audiology referral clinics), patients tested in wards because of poor immunity and isolation (33%, *n* = 4 oncology units; 57%, *n* = 4 audiology referral clinics), which results in environmental noise (83%, *n* = 10 oncology units; 86%, *n* = 6 audiology referral clinics) and unfavourable testing conditions and financial considerations (25%, *n* = 3 oncology units). An open-ended response from the private oncology units was ‘The patients are put through a lot very quickly and it is extremely stressful to them. Cost is a big factor’.

As it is clear that there is a lack of ototoxicity monitoring protocols followed, 83% (*n* = 10 oncology units; 86%; *n* = 6 audiology referral clinics) were in favour of a national ototoxicity monitoring protocol to be implemented in hospitals; however, 43% (*n* = 3 audiology referral clinics) indicated that they would modify the protocol to be suited to their setting. A national ototoxicity protocol may also assist with lobbying for equipment in hospitals (57%, *n* = 4 audiology referral clinics); however, 43% (*n* = 3 audiology referral clinics) were unsure if this would help. From the audiology referral clinics (*n* = 7), 57% (*n* = 4) were in favour of a novel approach to monitoring such as automated smartphone audiometry, 14% (*n* = 1) were not in favour and 29% (*n* = 2) were unsure. An open-ended response from the audiology referral clinics stated that there is ‘a need for mobile testing equipment’.

## Discussion

This survey is the first to report the national status of ototoxicity monitoring in cancer patients in the public and private healthcare sector in South Africa. Both in the private and public healthcare sectors, ototoxicity monitoring protocols are not followed. In the public sector, hearing tests are performed according to clinician referrals. Clinicians refer if patients complain about hearing-related problems. Some hospitals attempted to implement a strict protocol to see all qualifying chemotherapy patients; however, the constant rotation of doctors did not allow for a smooth working system between audiology and oncology. Awareness campaigns result in a temporary influx of referrals but do not remain consistent (Maru & Malky, 2018). In the private sector, patients mostly refer themselves. Often, by this time, a hearing loss is already noticeable and likely irreversible. Similarly, a study in the United States of America reported that the physicians differed in their approaches to ototoxicity monitoring, from habitual referrals to audiology, to relying on patient self-referral (Garinis et al., 2018).

The feedback from the private sector was that the oncology units do not give as much attention to hearing loss as they should. Oncology units claim that it is not a lack of awareness of ototoxicity but rather because of the cancer diagnosis, advanced disease, other oncologic emergencies, emotional, financial and physical constraints that are prioritised (Carrera, Kantarjian, & Blinder, 2018; Oun, Moussa, & Wheate, 2018). Although platinum-based treatment is an ototoxicity risk in itself and risk-prediction models for platinum-related ototoxicity, based on age and cumulative dose, have been developed, these models do not accurately predict risk for individual patients (Landier, 2016). Patient risks such as younger age (particularly <5 years) at the time of therapy, diagnosis of a central nervous system tumour, diminished renal function, rapid intravenous administration and treatment with multiple potentially ototoxic agents (Oun et al., 2018) are identified as increased risks for ototoxicity. The private oncology units are of the opinion that identifying a patient who has a high risk is more valuable than identifying just anyone on platinum-based treatments as hearing loss does not seem to be a main complaint in patients seen. This was also reported in a South African study, where oncologists reported that patients do not complain of the ‘subtle’ symptoms of cisplatin ototoxicity, such as tinnitus (Paken et al., 2020; Whitehorn et al., 2014).

This study indicated a comprehensive understanding of ototoxicity across all disciplines; however, there is limited familiarity with implementing ototoxicity monitoring and referral pathways and greater awareness amongst healthcare professionals is needed. These findings were similar to previous studies internationally and in South Africa stating that professionals involved in the care and management of cancer patients need to improve their awareness of ototoxicity and refer timeously for audiological evaluation (Landier, 2016; Paken et al., 2020; Steffens et al., 2014). All participants in this study stated that platinum-based chemotherapy can cause hearing loss, tinnitus and vestibular problems, which has a moderate to severe impact on daily life. This corresponds with findings in similar research performed in South Africa and internationally (Ganesan et al., 2018; Landier, 2016; Oun et al., 2018; Paken et al., 2020; Whitehorn et al., 2014). Cancer patients, however, undergo significantly variable ototoxicity monitoring, and practices range from no baseline testing and routine monitoring to some form of testing in some patients, which seems to be a common phenomenon in current ototoxicity monitoring practices (Ganesan et al., 2018; Paken et al., 2020). Although survival rates remain the priority in cancer treatment, there needs to be more emphasis on the importance of remaining side effects and long-term symptoms such as hearing loss and tinnitus (Pearson et al., 2019). As the survival rate increases and it becomes clear that there will be a life beyond cancer, QoL becomes increasingly important.

All audiology referral clinics in this study described appropriate ototoxicity protocols that should be followed, but implementation remains a challenge, despite the presence of substantial evidence supporting the significance of early identification of ototoxic-induced hearing loss (Ganeson et al., 2018; HPCSA, 2018; Paken et al., 2020). Pure tone audiometry (PT), extended high frequency audiometry (EHF) audiometry and distortion product otoacoustic emissions (DPOAEs) were cited as the most crucial tests, as suggested in ototoxicity monitoring guidelines (Ganesan et al., 2018; Landier, 2016; Paken et al., 2020; Pearson et al., 2019). Although participants reported that vestibular problems may be caused by platinum-based chemotherapy, vestibular assessments are not typically included in monitoring protocols in both this study and internationally (Landier, 2016; Paken et al., 2020; Steffens et al., 2014). No widely accepted guidelines for vestibulotoxicity monitoring exist (Ganesan et al., 2018). The major challenge in vestibulotoxicity monitoring is the identification of these symptoms, which is only apparent when patients are mobilised and may often be incorrectly attributed to the patient’s debilitated state. Vestibular diagnostic procedures are also often impractical because of the patient’s compromised health status. Furthermore, because of the complex nature of the vestibular system, there is no single test that can identify vestibulotoxicity (Pearson et al., 2019).

Ototoxic testing was reported to continue 6–12 months post-treatment, with some suggesting follow-up for an entire person’s lifespan. Existing protocols suggest 6 months post-treatment and annually for at least 10 years (Landier, 2016; Pearson et al., 2019; Steffens et al., 2014). More than half of participants in this study indicated that patients were uninformed about ototoxic effects of chemotherapy. Research suggests that oncologists and nurses should be the custodians for providing this information (Paken et al., 2020; Pearson et al., 2019). A multidisciplinary team and patient-centred approach to ototoxicity is essential as effective communication amongst healthcare professionals and greater insight of information about adverse effects and monitoring is needed (Ganesan et al., 2018; Landier, 2016; Pearson et al., 2019). Monitoring outcomes are believed to influence dosage and treatment choices, result in otoprotective agents being prescribed, and it ensures follow-up appointments and frequent visits to the audiologist. This is in agreement with the purpose of ototoxicity monitoring protocols (Konrad-Martin et al., 2018; Landier, 2016; Maru & Malky, 2018; Pearson et al., 2019; Steffens et al., 2014).

The most prominent challenges reported by participants in this study were referral system, environmental noise, multidisciplinary teamwork, lack of equipment, staff availability and the often compromised status of cancer patients (Konrad-Martin et al., 2018). More than half of audiology referral clinics in this study were in favour of a novel approach to ototoxicity monitoring. Considering the challenges identified in ototoxicity monitoring, the integration of mobile health (mHealth) tools such as smartphone audiometry is a novel approach, which can improve the effectiveness and efficiency of ototoxicity monitoring in cancer patients. mHealth tools have proved to be effective in primary healthcare settings (Sandström, Swanepoel, Myburgh, & Laurent, 2016) and infectious disease clinics (Brittz, Heinze, Mahomed-Asmail, Swanepoel, & Stoltz, 2019); however, applications specifically for ototoxicity monitoring in cancer patients require further investigation. An mHealth hearing screening application with automated test sequences, integrated noise monitoring, data capturing and data sharing (Sandström et al., 2016; Yousuf Hussein et al., 2016) makes asynchronous ototoxicity monitoring possible, which will minimise the already overburdened schedule of cancer patients as monitoring can take place during in- or out-patient chemotherapy treatments.

## Conclusion

There is significant discrepancy in how ototoxicity monitoring is conducted across South Africa in both the private and public sectors, and implementation of a national ototoxicity monitoring protocol may improve audiological outcomes for patients receiving ototoxic chemotherapy. Health Professions Council of South Africa (2018) ototoxicity monitoring guidelines have been developed and should be used as a guide when implementing ototoxicity monitoring programmes.

Furthermore, effective scheduling and test location are key to a successful monitoring programme. Finally, the need to simplify ototoxic monitoring of hearing and vestibular function to reduce test time and make it less stressful and tiresome on the patient should be considered. Ototoxic monitoring programmes need to become standard of care for all patients receiving treatment with ototoxic medications. Although a multidisciplinary team approach is vital, audiologists must take the lead to implement programmes that are thorough, efficient and accurate and based on patient-centred care. Audiologists need to be proactive and develop exceptional working relationships with oncologists and nursing staff within the oncology units, ensuring that appropriate referrals are made for ototoxicity monitoring. Including ototoxicity monitoring in the oncology treatment programme may also limit the overwhelming costs involved in oncology treatment.

A deeper understanding of how long-term toxicities, such as hearing loss, tinnitus and vestibular dysfunction, can affect QoL, needs to be incorporated into clinical practice for audiology referral centres and oncology units. By raising awareness, the risk of these long-term effects being overlooked will reduce. Once enrolled in ototoxicity monitoring, cancer patients ought to be guided through the treatment journey and can be provided with pertinent and individualised support and intervention for hearing loss, tinnitus and vestibular dysfunction.
